# Predicting grain growth kinetic in steels using machine learning and XAI for mechanical properties

**DOI:** 10.1371/journal.pone.0341053

**Published:** 2026-01-16

**Authors:** Selim Demirci, Durmuş Özkan Şahin, Sercan Demirci, Mehmet Masum Tünçay, Moataz M. Attallah

**Affiliations:** 1 Marmara University, Faculty of Engineering, Department of Metallurgical and Materials Engineering, Istanbul, Turkey; 2 University of Birmingham, School of Metallurgy and Materials, Birmingham, United Kingdom; 3 Ondokuz Mayıs University, Faculty of Engineering, Department of Computer Engineering, Samsun, Turkey; Pacific Northwest National Laboratory, UNITED STATES OF AMERICA

## Abstract

Understanding and controlling grain growth kinetics in steels is crucial for optimizing mechanical properties during thermomechanical processing. However, traditional empirical models often fail to account for the complex, nonlinear interactions between alloying elements and processing parameters. In this study, we introduce a novel machine learning (ML) based framework that predicts austenitic grain growth behaviour directly from chemical composition and process conditions, utilizing a comprehensive dataset of 1039 experimentally validated samples. Among various algorithms tested, the XGBoost model demonstrated exceptional predictive capability, achieving an R^2^ value of 0.9728 after hyperparameter optimization. Feature selection methods (Pearson correlation, CfsSubset, ReliefF) and SHAP-based explainable AI analyses were employed to identify the most influential parameters, revealing temperature, initial grain size, and holding time as dominant factors. Experimental validation was conducted on 316L stainless steel samples annealed at 1100 °C. The predicted grain sizes showed strong agreement with experimental measurements, and the observed hardness variations followed the expected Hall–Petch behaviour. This study demonstrates the first integrated ML and experimental approach for predicting grain growth kinetics in steels, offering a powerful tool for alloy design and process optimization. Future work will extend this framework to additional process variables and alloy systems.

## 1. Introduction

Steel has long been a fundamental material in engineering and industrial applications due to its excellent mechanical properties, affordability, and versatility [1–3]. As an alloy primarily composed of iron and carbon, steel often includes additional elements such as manganese, chromium, nickel, and vanadium to enhance properties like strength, toughness, corrosion resistance, and thermal stability [4–6]. The mechanical behaviour of steel varies significantly based on its composition and processing conditions, making it indispensable in industries such as automotive, aerospace, construction, and energy [7–11]. Given its wide range of applications, optimizing steel’s mechanical properties is crucial. These properties can be tailored through various strengthening mechanisms, including solid solution strengthening and thermomechanical processes such as heat treatment, cold working, and annealing [12]. Among these factors, grain size plays a key role in determining steel’s mechanical strength. The Hall-Petch relationship demonstrates that smaller grain sizes increase strength due to the increased number of grain boundaries acting as obstacles to dislocation motion [13]. However, grain growth is an inevitable process in metals, as polycrystalline materials naturally seek to reduce their total grain boundary energy through coarsening. In steels, this phenomenon is often associated with the growth of the austenite (γ) phase grains, which can significantly influence the final microstructure and mechanical performance. If left uncontrolled, excessive grain growth can lead to a decline in mechanical strength and overall performance. During the cooling process, austenite grain boundaries serve as nucleation sites for phases such as ferrite, cementite, bainite, and martensite. When the austenite grain size is small, the number of nucleation sites increases, leading to a finer grain structure in the final phase [14]. Therefore, a thorough understanding of grain growth kinetics particularly in the austenite phase is essential for optimizing the microstructure of steel and maintaining its desired mechanical properties [15,16]. The rate of austenite grain growth depends on factors such as temperature, time, initial grain size, and the presence of solute elements and second-phase particles that inhibit boundary motion. The kinetics of austenitic grain growth are generally described by the empirical Equation (1) for isothermal grain growth, which defines the evolution of grain size during isothermal heating [17,18].


$$D^{n}-D_{\text{0}}^{n}=kt$$ (1)

where D represents the average grain diameter, D_0_ is the initial grain diameter, t denotes the soaking time at a given temperature, n and K are the growth exponent and rate constant, respectively. In addition to empirical formulations, the evolution of grain size in steels has been extensively studied through classical and computational approaches. Traditional theoretical models, such as the Burke–Turnbull parabolic growth law and Hillert’s mean-field theory, describe grain coarsening through curvature-driven grain boundary motion and provide the foundation for understanding thermal grain growth behaviour [19,20]. More advanced numerical techniques including Monte Carlo Potts simulations and phase-field modelling enable the description of complex mechanisms such as abnormal grain growth, solute drag, and particle-boundary interactions that cannot be captured by simple analytical equations [21,22]. These modelling approaches collectively form the basis of modern grain evolution theory and highlight the multi-scale nature of grain growth phenomena in steels. The growing field of data-driven microstructure prediction further builds on these foundations, motivating the development of machine learning (ML) based strategies for modelling grain evolution under diverse processing conditions. There have been various methods to control grain size in steel, including alloying strategies and advanced thermomechanical processing techniques [23]. One of the method is utilization microalloying elements like Nb, Ti, and V form fine precipitates like carbides, nitrides, and carbonitrides acting as effective grain boundary pinning agents that inhibit grain growth [24]. For instance, Zhu et al. [25] investigated the austenite grain growth for Nb and Al microalloyed 20MnCr steels. They found that NbC precipitates inhibit grain growth by stabilising grain boundaries more effectively than AlN precipitates. Xu et al. [26] investigated the effect of heating temperature and dwell time on austenite grain growth in SCM435 steel. A new model was proposed to accurately predict the average austenite grain size of SCM435 steel during heating, showing strong consistency with experimental data and improving upon the limitations of the classical Sellars model. Despite extensive experimental studies, predicting austenite grain growth behaviour remains challenging due to the complex interplay of composition, processing parameters, and microstructural interactions. Traditional empirical models often fail to capture these intricate dependencies, necessitating extensive experimental calibration for each steel grade. In this context, ML presents a powerful tool for uncovering hidden patterns within large experimental datasets and predicting microstructural evolution with high accuracy [27,28]. The goal of the ML model is to establish a functional relationship between inputs and outputs, represented as *y = f*_*model*_ (*x*_*i*_*)*. Various algorithms are employed to optimize the model’s parameters, ensuring that the predicted output *f*_*model*_ *(x*_*i*_) closely approximates the true value [29,30]. By integrating experimental data with ML models, researchers can establish quantitative relationships between alloying elements, heat treatment parameters, and resulting grain structures. This data-driven approach has the potential to revolutionize steel processing by minimizing trial-and-error experimentation and optimizing manufacturing techniques for improved performance. To the best of our knowledge, there is no research on utilizing ML for predicting grain growth kinetics in steel based on thermomechanical processing conditions and alloy composition. Unlike previous studies that primarily focused on empirical models or mechanical property predictions, this work presents the first comprehensive ML-based framework aimed specifically at predicting austenitic grain growth kinetics in steels. By combining an extensive dataset covering a wide range of chemical compositions and thermomechanical parameters with advanced feature selection techniques and explainable artificial intelligence (XAI) methods, this study establishes a direct link between input parameters and grain size evolution. Furthermore, the integration of experimental validation through 316L stainless steel annealing tests ensures the robustness and real-world applicability of the proposed model. The results demonstrate that ML, particularly the XGBoost algorithm, can achieve highly accurate predictions (R^2^ = 0.9728), providing a transformative tool for accelerating steel design and optimizing processing routes with minimal experimental effort.

## 2. Methodology and material

**Fig 1:**
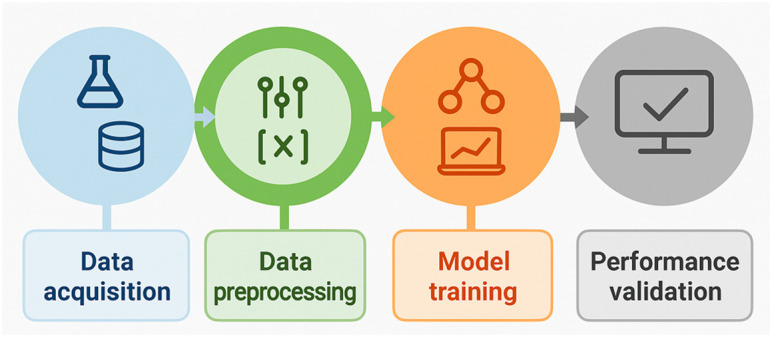
Schematic diagram of the machine learning (ML) process.

This research focuses on uncovering the relationship between the chemical composition of steels and the thermomechanical processing conditions that govern grain growth kinetics and final grain size. To achieve this, a machine learning-based framework was developed, encompassing four sequential phases: comprehensive data acquisition, data preprocessing, model training, and rigorous performance validation, as illustrated in Fig 1.

### 2.1. Data acquisition

For the purposes of this study, the dataset was obtained by conducting a thorough survey of existing experimental literature, regarding steels with a wide range of compositions and processing conditions. The data was manually gathered from well-established scientific platforms, including but not limited to Elsevier, Springer, and Wiley [1,2,16,17,23,31–67]. The dataset consists of 1039 samples in total and comprehensive statistical information in terms of both inputs (features) and outputs is given in Table 1. Input variables can be divided into two main groups: chemical compositions of steel (e.g., Fe, C, Cr, Mn, Si, etc.) and thermomechanical parameters such as temperature (°C), holding time (h), initial grain size (D_0_, μm), final grain size (D, μm), growth exponent (n), rate constant (k) and % cold rolling (R). The final grain size (μm) is denoted as an output in the problem. The output variable D exhibits a wide distribution between 3.99 μm and 990 μm. The wide range of the output value D indicates that the problem could be quite difficult. This problem may require the consideration of multidimensional and potentially non-linear relationships. In general, the dataset is diverse in both chemical and physical parameters and allows for a statistically meaningful analysis.

**Table 1:** Descriptive statistics of the inputs and output of various parameters in the dataset.

Features (Input)	Variable	Max	Min	Mean	Std
Temperature (°C)	Numeric	1350	600	1058.724735	117.6714314
Time (h)	Numeric	48	0.00027	2.480402493	3.796455761
D_0_ (μm)	Numeric	446.9	0	18.8144795	50.60096027
n	Numeric	44	0.72	3.949349374	3.931787151
lnk	Numeric	205.1	−0.35	14.41307517	16.68692264
R	Numeric	95	0	4.908565929	18.99453105
Fe	Numeric	99.709	57.794	89.15704878	13.34812718
C	Numeric	1.03	0.005	0.2909111646	0.2909667863
Cr	Numeric	22.06	0	3.961348412	5.774009231
Mn	Numeric	30.8	0	1.673525505	5.048520458
Si	Numeric	2.562	0	0.3590105871	0.4516603488
Al	Numeric	9.2	0	0.2915514918	1.541285414
Nb	Numeric	1.4	0	0.07351799808	0.2086532827
V	Numeric	0.64	0	0.04103387873	0.1188772341
Mo	Numeric	5.56	0	0.6977083734	1.335771647
Ni	Numeric	22	0	2.190352262	4.65171283
P	Numeric	0.059	0	0.007594610202	0.01016320434
S	Numeric	0.069	0	0.006339172281	0.01077102531
N	Numeric	0.384	0	0.005806564004	0.02574829995
Ti	Numeric	0.5	0	0.0178719923	0.0593969532
Cu	Numeric	1.62	0	0.05538238691	0.2054873846
B	Numeric	0.001	0	3.92E-05	0.0001722168956
W	Numeric	1.7	0	0.1780943215	0.4564409806
Co	Numeric	14.53	0	0.9833493744	3.424367506
**Output**	**Variable**	**Max**	**Min**	**Mean**	**std**
D (μm)	Numeric	990	3.99	86.96410972	114.4690775

### 2.2. Data preprocessing

In modelling the grain size of steels using machine learning, it is essential to account for a wide range of variables, including both chemical compositions and thermomechanical processing parameters such as temperature, time, and deformation. These variables are often collected from diverse sources and may contain missing values, inconsistencies, or differences in scale and units. Feeding such raw data directly into a model can lead to incorrect learning patterns or physically invalid predictions. Moreover, not all input variables have a significant influence on the final microstructure, and including irrelevant features may reduce model performance. Therefore, the data preprocessing comprising data cleaning, normalization and unit consistency is a critical step to ensure that the model produces reliable, interpretable, and scientifically meaningful results. This process directly enhances the generalizability and accuracy of data-driven predictions in materials science applications. In this context, normalization is applied to improve model robustness.

#### 2.2.1. Normalization.

Normalization is a crucial preprocessing step for ensuring balanced model training, especially when input variables have different scales. In this study, Min-Max normalization was applied to rescale all features between 0 and 1, as shown in Equation (2) [68–70].


$$x_{normalized}=\frac{x-x_{min}}{x_{max}-x_{min}}$$ (2)

where x is the actual value, and $x_{min}$ and $x_{max}$ are the minimum and maximum values of the feature, respectively. Normalization prevents models from being biased toward variables with larger magnitudes and enhances the performance of especially distance-based algorithms. This step is essential given the wide range of parameters such as temperature, holding time, and alloying element concentrations in the dataset.

### 2.3. Data splitting

After the normalisation process, the dataset was divided into 80% training and 20% testing in order to provide an objective evaluation of the model performance. This distinction makes it possible for ML algorithms to use only training data in the learning process and then evaluate the generalisation ability of the modelled structure on test data. In order to ensure that all algorithms operate on the same data split and to increase the comparability of the results, the random_state parameter is fixed in the data splitting process. Fixing this parameter guarantees experimental reproducibility by preventing variations due to randomness. Thus, by training and testing different algorithms on the same samples, the reliability of the performance metrics obtained and the consistency of the comparative analyses are increased.

### 2.4. Machine learning (ML) algorithms

In this study, various supervised ML algorithms are applied to predict the dependent variable D in the dataset. The methods used consist of both parametric and non-parametric algorithms, representing different modelling approaches. In this context, Adaboost [71], Decision Tree (DT) [72], Gaussian Process Regression (GPR) [73], K-Nearest Neighbors (KNN) [74], Linear Regression (LR) [75], Multi-Layer Perceptron (MLP) [76], Random Forest (RF) [77], Support Vector Regression (SVR) [78] and XGBoost [79] algorithms were used through the scikit-learn library. The selected ML algorithms were applied with default hyperparameter settings to obtain baseline model performances. This baseline model is intended to observe how the algorithms interact with the data under the default configurations. However, it is very useful to optimise the hyperparameter settings to improve model performance and to obtain better generalisation capability. Within this context, a systematic hyperparameter search was performed for each algorithm using the GridSearchCV method in the scikit-learn library. GridSearchCV selects the parameter set that provides the best performance by evaluating the specified parameter combinations with the cross-validation method. Thus, the configuration of each model is tailored specifically to the data set, reducing the risk of overfitting and providing more balanced and effective prediction models. Table 2 shows the hyperparameters obtained with the GridSearchCV approach.

**Table 2:** Selected parameters of GridSearchCV approach.

Algorithms	Selected hyperparameters
**Adaboost**	‘learning_rate’: 0.01, ‘n_estimators’: 225
**DT**	‘max_depth’: None, ‘min_samples_leaf’: 1, ‘min_samples_split’: 2, ‘random_state’: 42
**GPR**	‘alpha’: 0.01, ‘n_restarts_optimizer’: 0 (As in the default parameter)
**KNN**	‘algorithm’: ‘auto’, ‘n_neighbors’: 1, ‘p’: 1, ‘weights’: ‘uniform’
**LR**	‘fit_intercept’: True (As in the default parameter)
**MLP**	‘activation’: ‘relu’, ‘alpha’: 0.001, ‘hidden_layer_sizes’: (100, 100), ‘learning_rate’: ‘constant’, ‘solver’: ‘lbfgs’
**RF**	‘bootstrap’: True, ‘max_depth’: 20, ‘min_samples_leaf’: 1, ‘min_samples_split’: 2, ‘n_estimators’: 300
**SVR**	‘C’: 1.5, ‘epsilon’: 0.01, ‘gamma’: ‘scale’, ‘kernel’: ‘linear’
**XGBoost**	‘colsample_bytree’: 0.9, ‘gamma’: 0.2, ‘learning_rate’: 0.1, ‘max_depth’: 5, ‘n_estimators’: 300, ‘subsample’: 0.8

### 2.5. Feature selection

In this study, feature selection was performed to enhance model performance, reduce computational cost, and improve interpretability. Three different methods were applied: Pearson correlation coefficient, CfsSubset, and ReliefF [80,81]. Pearson correlation coefficient, *r*, measures the degree and direction of the linear relationship between two variables and is defined as given in Equation 3.


$$r=\frac{\sum_{i=\text{1}}^{n}\left(x_{i}-\overset{―}{x})(y_{i}-\overset{―}{y}\right)}{\sqrt{\sum_{i=\text{1}}^{n}{\left(x_{i}-\overset{―}{x}\right)}^{\text{2}}}\;\sqrt{\sum_{i=\text{1}}^{n}{\left(y_{i}-\overset{―}{y}\right)}^{\text{2}}}}$$ (3)

Variables with strong correlations to the target were selected, while weakly correlated or irrelevant features were excluded. The CfsSubset method evaluates subsets of features based on their predictive ability and redundancy, aiming to select inputs that are highly correlated with the target but minimally correlated with each other [82]. Its fitness criterion is shown in Equation (4).


$${merit}_{s}=\frac{k\cdot \;\overset{―}{r_{cf}}}{\sqrt{k+k(k-\text{1})\cdot \;\overset{―}{r_{ff}}}}$$ (4)

where k is the number of features, $\overset{―}{r_{cf}}$ is the average feature-target correlation, and $\overset{―}{r_{ff}}$ is the average feature-feature correlation. Finally, the ReliefF algorithm, particularly effective for complex, nonlinear problems, assigns weights to features based on their ability to differentiate instances with similar or different outputs [83]. The weight update rule is given in Equation (5) [84].


$$W[A]=W[A]-\frac{diff(A,\;x,H)}{m}+\frac{diff(A,x,M)}{m}$$ (5)

where W[A] is the weight of feature A, H and M are the nearest neighbours from the same and different classes (adapted for continuous targets), and mmm is the number of random samples. These three methods collectively provide complementary insights, improving feature selection robustness for predicting grain growth kinetics.

### 2.6. Evaluation of metrics

The models are assessed using three distinct evaluation metrics: Mean Absolute Error (MAE), Root Mean Square Error (RMSE), and R-squared (R^2^) in the following equations [70].


$$\begin{array}{c} MAE=\frac{\text{1}}{n}\sum {\mathrm{\backslash nolimits}}_{i=\text{1}}^{n}\left|y_{i}-{\hat{y}}_{i}\right| \end{array}$$ (6)

$$\begin{array}{c} RMSE=\sqrt{\frac{\text{1}}{n}\sum {\mathrm{\backslash nolimits}}_{i=\text{1}}^{n}{\left(y_{i}-{\hat{y}}_{i}\right)}^{\text{2}}} \end{array}$$ (7)

$$R^{\text{2}}=\text{1}-\frac{\sum_{i=\text{1}}^{n}{\left(y_{i}-{\hat{y}}_{i}\right)}^{\text{2}}}{\sum_{i=\text{1}}^{n}{\left(y_{i}-\overset{―}{y}\right)}^{\text{2}}}$$ (8)

Here $y_{i}$, ${\hat{y}}_{i}$, $\overset{―}{y}$ and n represent the actual value, the predicted value, mean value, and the number of observations, respectively.

### 2.7. Experimental studies

In this study, the austenitic stainless steel (316) is used to validate ML prediction. The chemical composition of the steels is given in the Table 3.

**Table 3:** Chemical composition (wt.%) of the austenitic stainless steel (316L).

Steel	C	Cr	Ni	Mn	Mo	Si	S	P	N	Fe
316L	0.02	19	12	1.9	2	0.4	0.001	0.002	0.06	64.617

The steels sample were heat treated at 1100 °C for 30, 45, and 60 minutes and cooled in air in order to observe grain growth. As-received and heat-treated samples were metallographically prepared by sequential grinding with SiC papers of 180, 240, 320, 400, 600, 800, 1000, and 1200 grit, followed by final surface polishing using a 0.05 µm alumina suspension. Then, all samples were cleaned in an ultrasonic bath with ethanol for five minutes. The etching of the austenitic stainless steel was performed by using Aqua Regia. The observation of the microstructures was carried out with an optical microscope (Olympus). The optical micrographs, captured at 20 × magnification, were analysed with ImageJ software to determine average grain size for 316 austenitic stainless steels. The hardness of each sample was measured utilizing a microhardness tester (FutureTech FM-ARS7000) using 100 g of load. The results were reported as an average of five measurements.

## 3. Results and discussions

### 3.1. Performances of ML algorithms

In this section, the performances of ML algorithms on the prediction of the output value are analysed and discussed. After the normalisation process is applied to the dataset, the dataset is divided into 80% training and 20% testing in order to provide an objective evaluation of the model performance. Firstly, the selected ML algorithms were applied with default hyperparameter settings and baseline model performances were obtained. Table 4 shows the results obtained using the predefined parameters of the ML algorithms in the sklearn library.

**Table 4:** Test results obtained from ML algorithms (using predefined parameters).

ML Algorithm	R^2^	RMSE	MAE
**Adaboost**	0.7852	61.6018	51.6792
**DT**	0.9152	38.7106	21.3065
**GPR**	0.7682	63.995	44.449
**KNN**	0.9021	41.5815	20.3712
**LR**	0.8056	58.6093	39.866
**MLP**	0.7945	60.2557	39.4829
**RF**	0.9424	31.8868	14.031
**SVR**	0.7623	64.8006	37.3978
**XGBoost**	0.9605	26.3923	12.7657

According to the modelling results with predefined parameters, significant differences were observed in the prediction performance of different ML algorithms on the test data as given in Table 4. The highest performance was achieved by the XGBoost algorithm, which, with R^2^ = 0.9605, RMSE = 26.39 and MAE = 12.77. Following XGBoost, the RF algorithm also provided high prediction. The RF algorithm showed a remarkable success with R^2^ = 0.9424, RMSE = 31.89 and MAE = 14.03. These results show that ensemble-based methods (especially tree-based models) are suitable for the considered problem and have strong predictive capacities. However, classical regression and distance-based algorithms showed more limited performance. For example, although the LR model has a reasonable explanatory power with R^2^ = 0.8056, the RMSE and MAE values (58.61 and 39.87, respectively) are higher, indicating that the model does not adequately capture complex patterns. Similarly, SVR and GPR algorithms also showed limited performance with relatively low R^2^ values (0.7623 and 0.7682) and high error metric values. In this context, ensemble and network-based algorithms, which can better model non-linear relationships and input interactions, stand out. However, it is very useful to optimise the hyperparameter settings to improve model performance and to obtain better generalisation capability. Within this context, a systematic hyperparameter search was performed for each algorithm using the GridSearchCV method in the scikit-learn library. Table 5 shows the results obtained using the GridSearchCV method in the scikit-learn library. As a result of the hyperparameter optimisation performed with the GridSearchCV method, a general improvement in the performance of ML models was observed.

**Table 5:** Test results obtained from ML algorithms (using GridSearchCV).

ML Algorithm	R^2^	RMSE	MAE
**Adaboost**	0.7915	60.6918	43.3559
**DT**	0.9228	36.9298	18.8681
**GPR**	0.7682	63.995	44.449
**KNN**	0.9029	41.4133	20.441
**LR**	0.8056	58.6093	39.866
**MLP**	0.8632	49.1516	31.9236
**RF**	0.9424	31.8923	13.7931
**SVR**	0.7639	64.5809	37.2893
**XGBoost**	0.9728	21.8908	10.9669

In particular, the XGBoost algorithm showed a significant performance improvement after hyperparameter adjustments, reaching R^2^ = 0.9728, RMSE = 21.89 and MAE = 10.97. These values indicate that the model is able to predict the output variable D with a very high accuracy and the error level is minimised. Similarly, the DT algorithm has also improved compared to its previous state with R^2^ = 0.9228, RMSE = 36.93 and MAE = 18.87 after optimisation. In contrast, some algorithms showed limited or no performance improvement. For example, performance metrics did not change despite hyperparameter optimisation in the GPR and LR models. Moreover, the MLP algorithm improved significantly after GridSearchCV, reaching R^2^ = 0.8632, indicating that the learning capacity of the model can be significantly increased with appropriate hyperparameters. In general, it has been shown that hyperparameter optimisation with GridSearchCV provides significant performance improvements especially in complex and parametric models and is a critical step in the model selection process. This reveals that algorithms can become significantly more efficient when configured with the right combinations of hyperparameters.

**Fig 2:**
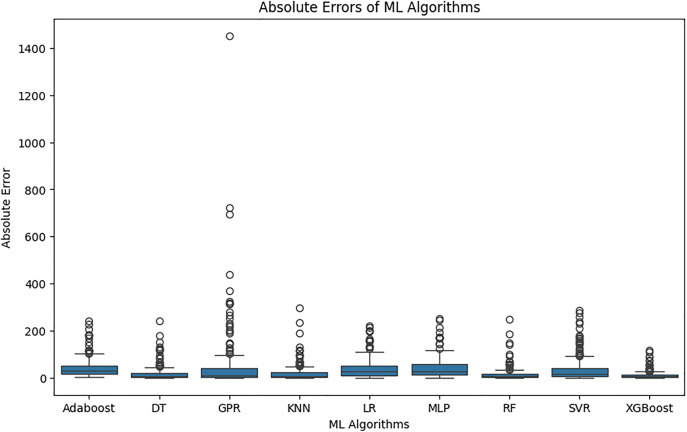
Boxplot of absolute error values of ML algorithms.

Fig 2 shows a boxplot plot to observe the absolute error between the prediction values of ML algorithms and the actual output values. The boxplot plot, which was created to examine the prediction performances obtained after hyperparameter optimisation in more detail, visually presents the absolute error distributions of ML algorithms in a comparative manner. In the Fig 2, especially the XGBoost algorithm has a low median error value and narrow interquartile range, indicating that this model produces forecasts with high accuracy and the error distribution is stable. Similarly, RF, DT and KNN models also show strong performance with relatively low error levels and limited outliers. On the other hand, the high number and value of outliers observed in the GPR algorithm shows that this model makes quite large errors in some cases and its predictions are not consistent. Overall, the findings in the Fig 2 are consistent with the numerical performance metrics and confirm that the XGBoost model is the most successful model in terms of both error level and stability of the error distribution.

**Fig 3:**
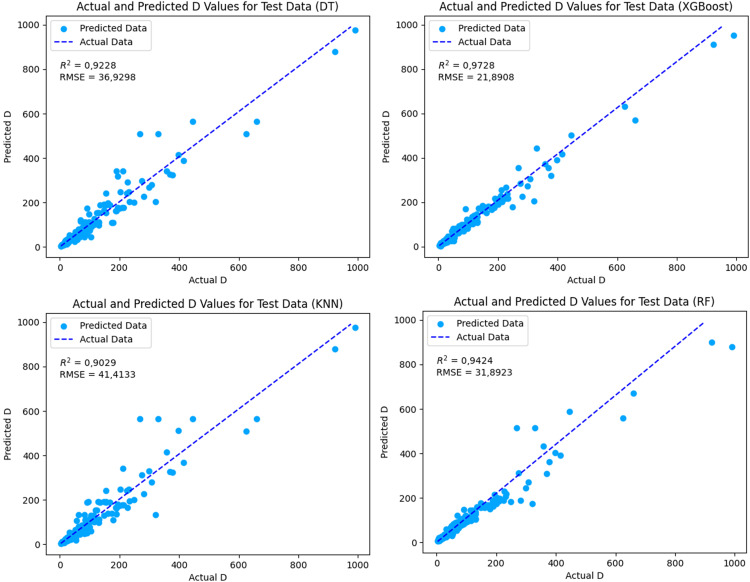
Actual and predicted D values for test data (respectively XGBoost, RF, DT and KNN).

Fig 3 shows the regression plots of the 4 most successful algorithms (XGBoost, RF, DT and KNN). Regression plots are very useful for comparing the accuracy of the models by visually presenting the relationship between predicted values and actual values. The four most successful algorithms, which are XGBoost, RF, DT and KNN models, were evaluated on the test data. The orange dots in the graphs represent the predictions and the red dashed line represents the ideal prediction state (actual = predicted). The XGBoost model showed a superior performance, with the point forecasts showing a distribution very close to the actual values and reaching a high coefficient of determination of R^2^ = 0.9728. The RF and DT models also have high explanatory power, the density of the predictions is distributed close to the linear. Although the KNN model exhibited a more dispersed structure than the other three models, it provided a high accuracy with R^2^ = 0.9029 and demonstrated reasonable generalisation success of the model. Overall, these graphs effectively visualise both the model performance and the error distribution, confirming that XGBoost performs well on this problem.

### 3.2. Effect of feature selection on the ML performances

**Fig 4:**
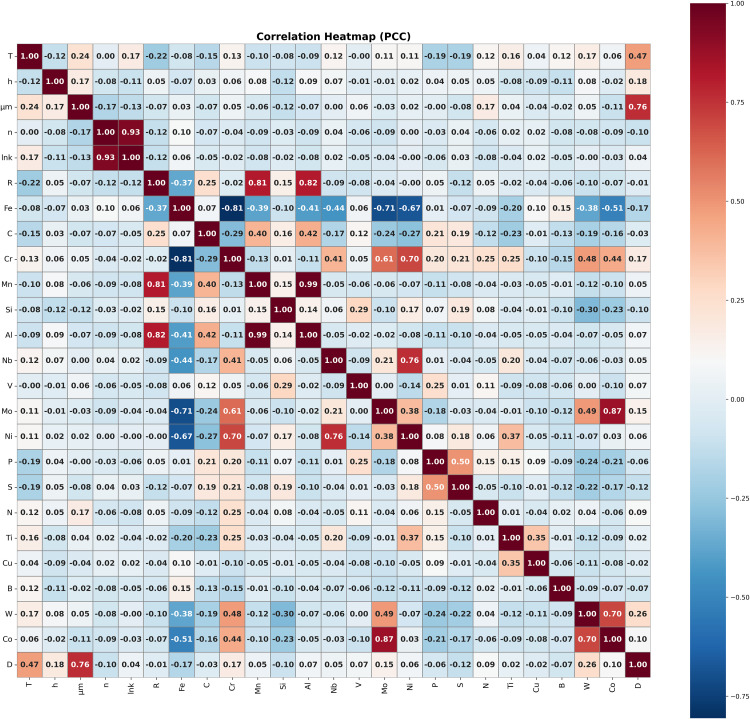
Pearson correlation heatmap for process parameters and grain size for this study.

Three different feature methods are applied to determine which inputs are more important for the regression problem. Firstly, the Pearson correlation analysis is performed to determine the linear relationships between the parameters analysed in the study. This method is preferred to quantitatively evaluate the direction and strength of the linear relationship between two continuous variables. The heat map given in Fig 4 visually presents the Pearson correlation coefficients between the variables in the dataset. Positive values of the correlation coefficient close to +1 indicate a strong linear relationship, while negative values close to −1 indicate a strong inverse relationship. Correlation coefficient values close to 0 indicate that there is no significant linear relationship between the variables.

Fig 4 shows the Pearson correlations between all variables and their relationship with the final grain size (D). As expected, the initial grain size (D_0_) has the strongest positive correlation with D (r = 0.76). This result clearly demonstrates that the final grain size of the initial microstructure is the most important determinant. In contrast, the holding time (h) (r = 0.18) and temperature (T) (r = 0.47) show only weak to moderate correlations with D. Therefore, these two variables alone should not be expected to strongly explain grain size; however, they still make measurable contributions to growth behaviour. The effect of chemical composition on D is generally weak. Among the alloying elements, tungsten (W), chromium (Cr), and molybdenum (Mo) have the highest positive correlation coefficients; however, these values are also quite low (r = 0.26, 0.17, and 0.15). Nevertheless, these trends are significant from a metallurgical perspective: W can increase grain boundary mobility by affecting grain boundary energy and diffusion, while Mo can alter grain growth at high temperatures through precipitation and solute drag effects [85–87]. However, low correlation coefficients indicate that the effects of these elements are not decisive on their own, but rather occur in conjunction with other variables. Although slight negative correlations were observed for iron (Fe), sulfur (S), and growth base (n), the very small values of these correlations (|r| < 0.1) indicate that their effects are negligible [88]. In general, it is understood that heating conditions, duration, and chemical composition must be evaluated together, and that grain size cannot be explained by a single parameter.

**Fig 5:**
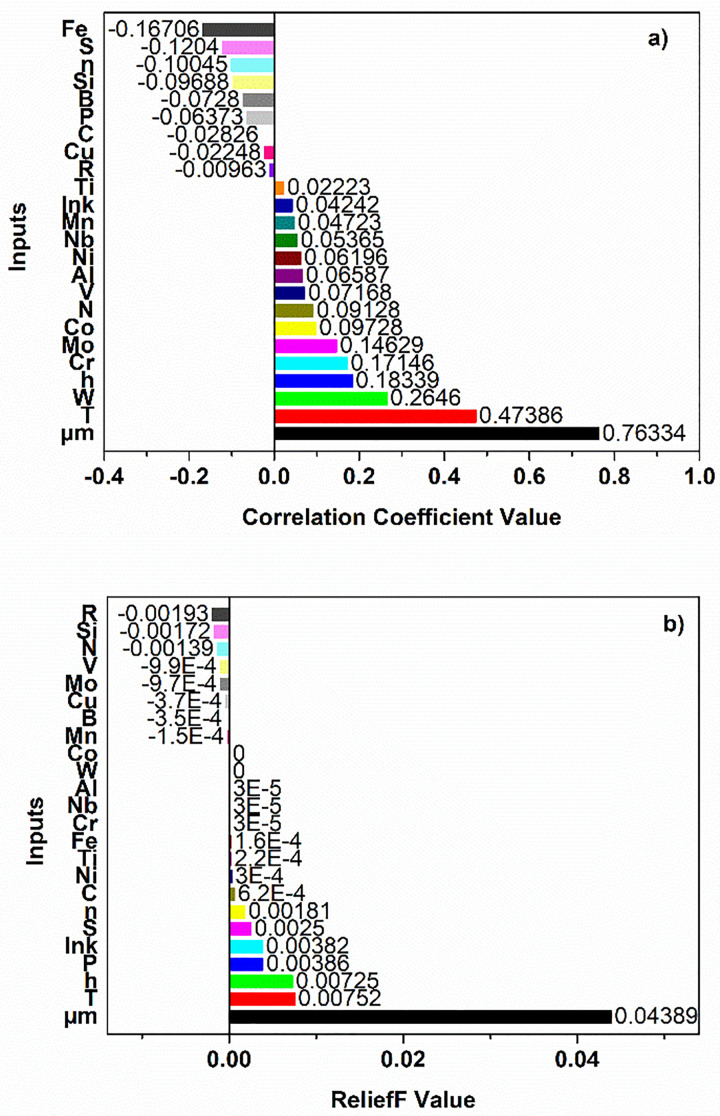
Feature importance ranking of input parameters affecting final grain size. (a) Features ranked by correlation coefficient values. (b) Feature importance ranking obtained using the ReliefF algorithm.

Fig 5 presents the ranking of the importance of the inputs in terms of their effect on the final grain size as a result of correlation analysis and ReliefF algorithm.

The first 6 inputs with the highest correlation coefficient values and 3 inputs with the lowest correlation coefficient values were selected, totalling 9 inputs as seen in Fig 5(a). This input subset consists of μm, T, W, h, Cr, Mo, Co, Fe, S and n. According to the ReliefF algorithm, the input importance ranking is given in Fig 5(b). According to the ReliefF algorithm, the number of nearest neighbours (k) value was found to be 10. A threshold value greater than 0.0002 was determined on the values given in Fig 5(b) and input subset selection was performed. As a result, 10 inputs were selected by ReliefF algorithm. These are μm, T, h, P, lnk, S, n, C, Ni and Ti. As for CfsSubset technique, merit of best subset was found as to be 0.777. Seven features were automatically selected by CfsSubset. These attributes are T, h, μm, Fe, V, P and W. Table 6 shows the regression results obtained using feature selection methods. The performance results obtained after feature selection reveal that the inclusion of certain variables in the model leads to significant improvements in prediction accuracy. As a result of modelling using input sets selected by correlation coefficient, CfsSubset and ReliefF methods, the highest performance was generally obtained by the XGBoost algorithm. In particular, modelling with CfsSubset method using 7 inputs yielded R^2^ = 0.975, RMSE = 21.00 and MAE = 10.59, indicating that even a small number of carefully selected inputs can provide high accuracy. This has the advantage of reducing the complexity of the model, reducing the computational cost and preventing overlearning.

**Table 6:** Effect of feature selection on output prediction performance.

Feature Selection Techniques	ML Algorithm	Metrics		
		R^2^	RMSE	MAE
Correlation Coefficient (9 Features)	**DT**	0.8808	45.8869	24.3112
	**KNN**	0.9087	40.1499	18.9846
	**MLP**	0.7887	61.0972	41.0543
	**RF**	0.9371	33.3355	15.3248
	**XGBoost**	**0.961**	26.2198	11.8417
CfsSubset (7 Features)	**DT**	0.8943	43.2146	23.6615
	**KNN**	0.8842	45.2296	25.139
	**MLP**	0.7891	61.0421	40.4584
	**RF**	0.9352	33.8286	16.101
	**XGBoost**	**0.975**	21.0036	10.5903
ReliefF (10 Features)	**DT**	0.9155	38.6361	21.18
	**KNN**	0.902	41.6089	20.3366
	**MLP**	0.8422	52.7906	34.0108
	**RF**	0.9354	33.7837	16.4203
	**XGBoost**	**0.9679**	23.7881	11.5792

The RF and DT algorithms have also largely maintained their high performance after feature selection, and in some cases even improved it. On the other hand, the MLP algorithm performed worse than all three methods, indicating that MLP is more sensitive to the selected input combinations. In general, the ReliefF method provided more balanced results, especially in terms of DT and MLP algorithms. The findings show that model performance can be improved with appropriate feature selection, and this effect becomes even more pronounced when used with powerful algorithms such as XGBoost. In this context, feature selection makes an important contribution to both model optimisation and interpretability. When the performance of the KNN algorithm after feature selection is analysed, certain differences are observed in the results depending on the methods used. The R^2^ = 0.9087, RMSE = 40.15 and MAE = 18.98 values obtained with 9 inputs selected by the correlation coefficient method reveal that the KNN algorithm shows a very successful prediction performance. These results show that KNN can be a highly powerful local prediction model when fed only with meaningful inputs. However, when using the 7 inputs selected with the CfsSubset method, a decrease to R^2^ = 0.8842 was observed and the error metrics also deteriorated significantly (RMSE = 45.23; MAE = 25.14). This shows that the inputs selected with the CfsSubset method are not sufficiently compatible with the distance-based structure of the KNN model. On the other hand, achieving R^2^ = 0.902 with 10 inputs selected with the ReliefF method and maintaining a successful prediction performance with low error metrics (RMSE = 41.61, MAE = 20.34) indicate that this method provides more suitable input combinations for KNN model. In general, it can be said that the KNN algorithm is sensitive to feature selection and especially the correlation coefficient and ReliefF based selections positively affect the performance of this algorithm. This is evidenced by the fact that results close to these results are obtained with the KNN algorithm without feature selection.

### 3.3. Interpreting results with explainable artificial intelligence (XAI)

**Fig 6:**
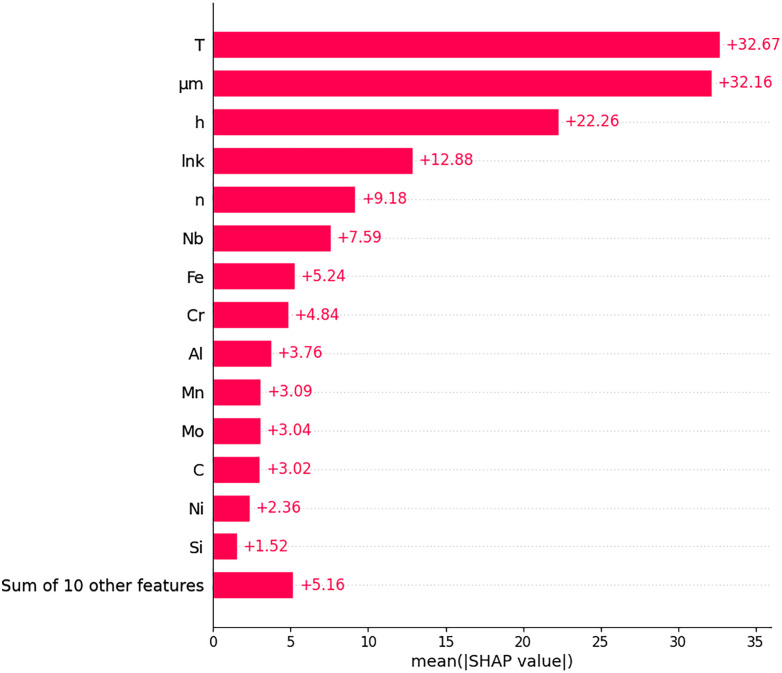
Feature importance ranking based on mean SHAP values (mean|SHAP|) calculated for the XGBoost model.

**Fig 7:**
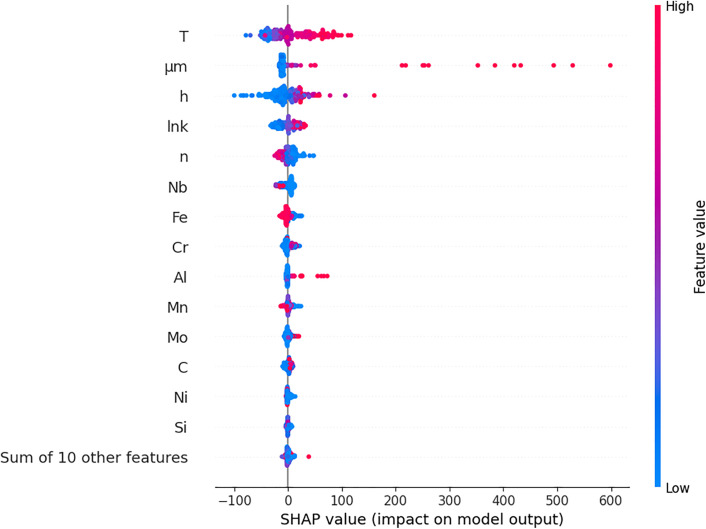
SHAP beeswarm graph of XGBoost algorithm.

In this subsection, the SHAP analysis was performed on XGBoost since XGBoost was the most successful algorithm in the experiments. This analysis not only increases the explainability of the model but also reveals which inputs are really effective in a concrete way. Figs 6 and 7 demonstrate the graphs obtained from the SHAP analysis. These graphs show in detail the input effects of the XGBoost model on the output variable D at both global and local level. The bar graph in Fig 6 shows the average contribution (mean |SHAP| value) of the inputs to the model.

According to Fig 6, the three most effective inputs are T, μm and h, respectively. These three physical parameters are the variables that make the highest contribution to the output of the model on average, and it is understood that the model considers these factors primarily on the output variable. In particular, T and μm have very close SHAP contribution values, indicating that these two variables are effective with similar weights. The fact that chemical and structural properties such as lnk, n, Nb, Fe and Cr are among the following variables reveals that not only physical but also alloy composition and microstructure characteristics make a significant contribution to the model. The beeswarm plot given in Fig 7 reflects the direction and intensity of the input values on the prediction for each sample. The graph in Fig 7 visualises not only the magnitude but also the direction of the effect of the inputs on the model.

Here, pink tones represent high values and blue tones represent low values. For example, high values of variables such as T and μm generally have a positive effect on the forecast, while low values have a negative effect as seen in the Fig 7. This shows that the model tends to increase the D output with higher values of T and μm. Similarly, higher values of alloying elements such as Nb and Cr also contribute positively to the model, but their effects are more limited compared to the physical parameters. As a result, SHAP analyses reveal in detail not only which inputs are important but also how these inputs shape model decisions. In this respect, it is seen that the XAI approach is an indispensable component for model interpretability as well as model accuracy. The findings support that the data preprocessing and engineering information are selected correctly and the model makes decisions in accordance with the physical reality. When the results obtained by feature selection methods and SHAP-based explainability analysis are evaluated together, significant overlaps are observed between the variables that the model inputs importance in the learning process and the selected inputs. In particular, the μm, T and h inputs were selected by both Correlation Coefficient, CfsSubset and ReliefF methods. Also, the μm, T and h inputs stood out as the top three inputs that contributed the most to the SHAP analysis. This strongly confirms that these physical parameters have a determining effect on the output variable D of the model. Moreover, the inclusion of variables such as Fe, W, n, P, and S in the model through different feature selection approaches, along with their notable contributions in the SHAP analysis, indicates that both statistical and explainability-based analyses yielded consistent results. In contrast, features like lnk, C, Ni, and Ti, which were selected only by the ReliefF method, exhibited lower SHAP values, implying a relatively minor contribution to the model’s predictions on a sample-by-sample basis. In general, a high level of agreement was observed between the results of the feature selection methods and the findings of the SHAP analysis, which both increases model reliability and supports the accuracy of the feature selection strategies.

### 3.4. Experimental verification

**Fig 8:**
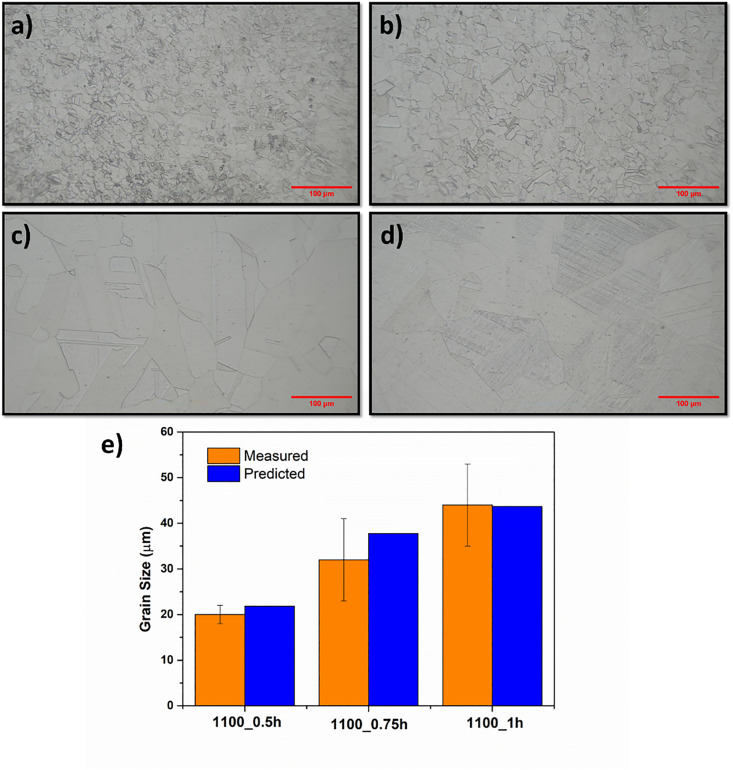
The OM microstructure of austenite grain in experimental steel heated at 1100 °C for different temperature (a) as-received; (b) 30 min; (c) 45 min; (d) 60 min; (e) comparison of predicted grain size with experimental results.

Fig 8 illustrates the optical microstructure of the 316 steels after heat treatment at temperature of 1100 °C with a different holding times. As shown in Fig 8, these micrographs of steels enable relatively precise identification of the austenite grain boundaries. Initially, small and dispersed grains are observed, while as the annealing time increases, the grains become larger and the grain boundaries become more pronounced. This indicates that grain growth occurs with the activation of diffusion mechanisms at high temperature. The microstructure of steels shows the equiaxed grains. The average grain sizes of the austenite phase of 316 stainless steel are estimated 20 μm, 32 μm and 44 μm with increasing time, respectively. The obtained grain sizes were good agreement with literature [69]. The predicted and measured grain size of steels after heating process are shown in the Fig 8(e). The grain sizes were predicted by using XGBoost algorithm which exhibited best performances. As seen in Fig 8(e), the model is able to successfully predict grain growth, which shows the reliability of the prediction system developed or used.

Moreover, the Hall–Petch equation describes the relationship between a material’s hardness and its grain size as expressed:


$$H=H_{\text{0}}+k_{y}\times \frac{\text{1}}{\sqrt{d}}$$

in which H₀ refers to the inherent hardness of the alloy, *d* stands for the mean grain size, and k_y_ represents the Hall–Petch constant [89]. The hardness results of samples heated at 1100 °C were found to be 162 HV, 160 HV and 151HV with increasing holding time, respectively. This behaviour is attributed to the fact that steels with finer grain sizes possess a greater number of grain boundaries, which act as barriers to dislocation motion. As a result, dislocations require more energy to cross these boundaries due to variations in atomic orientation. The trend observed follows the Hall-Petch relationship, where the hardness of annealed steel decreases as the grain size becomes larger [90].

### 3.5. 95% confidence interval estimation using quantile XGBoost

**Fig 9:**
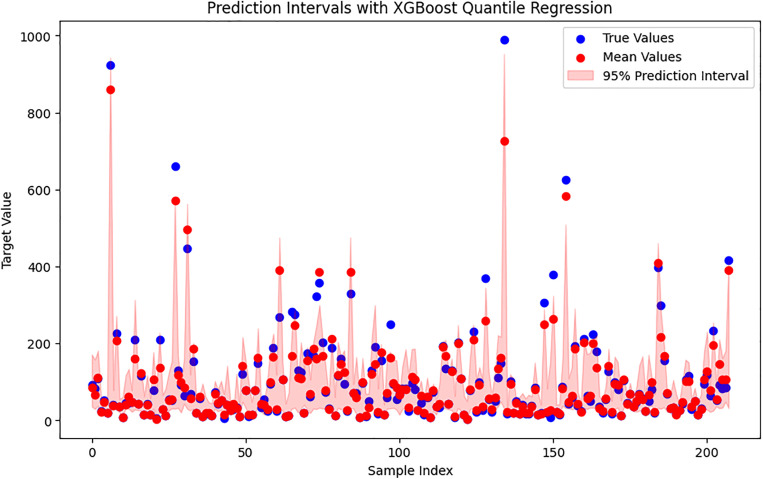
XGBoost-based quantile regression results on the test dataset.

To estimate the predictive uncertainty of the XGBoost model, we used quantile regression with the “reg:quantileerror” objective. In addition to the standard model that gives point predictions, we trained two extra models to estimate the lower (τ = 0.025) and upper (τ = 0.975) bounds of a 95% prediction interval. These quantile models were trained using the same hyperparameters as the main regressor, with the quantile level defined by the *quantile_alpha* parameter. For each test sample, the three models together provide the median prediction ${\hat{y}}_{\text{0}.\text{5}}\;$ and the uncertainty range $\left({\hat{y}}_{\text{0}.\text{025}},\;{\hat{y}}_{\text{0}.\text{975}}\right)$, which forms the 95% prediction interval. The accuracy of these quantile estimates was assessed using pinball loss. On the test set, the pinball loss values were 2.26 for the 2.5^th^ percentile, 7.39 for the median (50^th^), and 4.62 for the 97.5^th^ percentile. These values show that the quantile models provide a reasonable fit across the range of target values. Fig 9 illustrates the median XGBoost predictions along with the 95% prediction intervals and the corresponding experimental measurements. Experimental points are shown in blue, median predictions in red, and the shaded red region represents the uncertainty band. The interval successfully covers most of the experimental values and becomes wider in regions where the data show greater variability. This indicates that the quantile-based approach gives a meaningful picture of prediction uncertainty, which is important for evaluating the reliability of the model in microstructure design problems. The target variable ranges from about 4–990, which naturally affects quantile regression performance. The pinball loss at the median (τ = 0.5) is higher (7.3885) compared to the losses at the lower (2.2595) and upper (4.6231) quantiles. This suggests that the model has more difficulty predicting values near the center of the distribution, where the data are denser and more heterogeneous. In contrast, the lower pinball loss values at the extreme quantiles show that the model performs better at estimating the tails of the distribution. Overall, the consistent interval widths and relatively low pinball losses especially at the extreme quantiles confirm that the quantile XGBoost method captures prediction uncertainty effectively.

**Fig 10:**
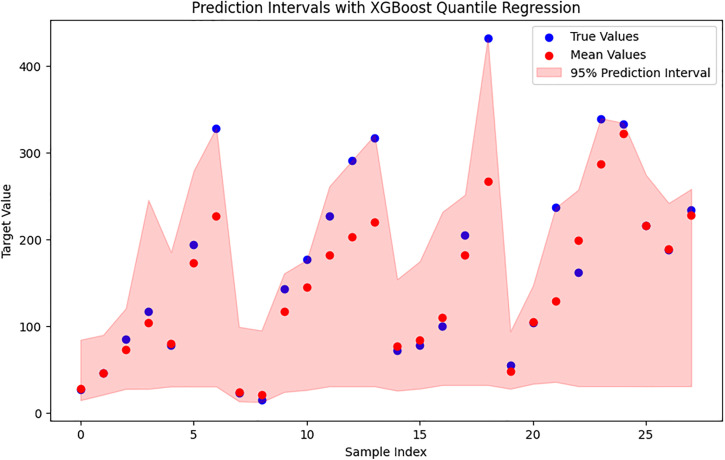
Quantile XGBoost 95% prediction intervals for the experimental verification samples.

The quantile regression results for the experimental validation samples show a different pattern compared to the test set as see in Fig 10. The pinball loss values were 3.61 for the 2.5^th^ quantile, 15.66 for the median (50^th^), and 1.24 for the 97.5^th^ quantile. The much higher loss at the median indicates that the model makes larger errors in predicting the central value for these samples. This is not surprising, because the validation data come from a separate experimental group with different processing conditions. In other words, these samples lie outside the distribution of the training data. Even though the median prediction is less accurate, the 95% prediction interval remains meaningful. The relatively low pinball loss values at the lower and upper quantiles show that the model still captures the uncertainty bounds quite well. As shown in the Fig 10, most experimental values fall within these intervals. The interval becomes wider in regions where the response varies more or where the validation conditions differ strongly from the training data. This behaviour suggests that the quantile XGBoost model adjusts its uncertainty estimates appropriately when facing unseen or shifted data. In summary, although the median predictions are less precise for out-of-distribution samples, the quantile-based intervals remain reliable and well-calibrated. This confirms that quantile regression is a useful tool for quantifying uncertainty and evaluating prediction reliability under new experimental conditions.

## 4. Conclusions

In this study, a comprehensive ML framework was developed to predict austenitic grain growth kinetics in steels based on alloy composition and thermomechanical processing parameters. A dataset comprising 1039 experimentally collected samples, covering a wide range of chemical elements (such as C, Mn, Cr, Ni, Mo) and processing conditions (temperature, holding time, initial grain size), was used to train and validate the models. Among various ML algorithms evaluated, the XGBoost model exhibited the best predictive performance, achieving an R^2^ value of 0.9728 after hyperparameter optimization. Feature selection techniques, including Pearson correlation, CfsSubset, and ReliefF, identified temperature, initial grain size, and holding time as the most influential parameters on grain growth behaviour. Explainable AI (SHAP analysis) further enhanced the interpretability of the model outputs. Experimental validation was performed on 316L stainless steel samples heat-treated at 1100 °C. The predicted grain sizes showed strong agreement with the measured values, confirming the model’s reliability. Furthermore, microhardness measurements demonstrated a consistent decrease in hardness with increasing grain size, aligning with the Hall–Petch relationship. This correlation between grain size and hardness not only validates the grain growth predictions but also emphasizes the critical role of microstructure control in mechanical property optimization. Overall, this study represents a novel integration of machine learning, feature engineering, and experimental validation for predicting grain growth kinetics in steels. The proposed framework significantly reduces the need for extensive experimental efforts and offers a powerful tool for accelerating alloy design and process optimization. Future studies will focus on expanding the model by incorporating additional processing variables, such as cooling rate and strain, and extending its application to other alloy systems beyond steels.
